# Induction of Oxidative Stress and Antioxidative Mechanisms in *Arabidopsis thaliana* after Uranium Exposure at pH 7.5

**DOI:** 10.3390/ijms160612405

**Published:** 2015-06-02

**Authors:** Eline Saenen, Nele Horemans, Nathalie Vanhoudt, Hildegarde Vandenhove, Geert Biermans, May Van Hees, Jean Wannijn, Jaco Vangronsveld, Ann Cuypers

**Affiliations:** 1 Belgian Nuclear Research Centre (SCK•CEN), Biosphere Impact Studies, Boeretang 200, 2400 Mol, Belgium; E-Mails: nele.horemans@sckcen.be (N.H.); nathalie.vanhoudt@sckcen.be (N.V.); hildegarde.vandenhove@sckcen.be (H.V.); geert.biermans@gmail.com (G.B.); mvhees@sckcen.be (M.V.H.); jwannijg@sckcen.be (J.W.); 2 Hasselt University, Centre for Environmental Sciences, Agoralaan Building D, 3590 Diepenbeek, Belgium; E-Mails: jaco.vangronsveld@uhasselt.be (J.V.); ann.cuypers@uhasselt.be (A.C.)

**Keywords:** uranium toxicity, *Arabidopsis thaliana*, oxidative stress, gene expression, ascorbate-glutathione cycle

## Abstract

To evaluate the environmental impact of uranium (U) contamination, it is important to investigate the effects of U at ecologically relevant conditions. Since U speciation, and hence its toxicity, strongly depends on environmental pH, the present study aimed to investigate dose-dependent effects of U at pH 7.5. *Arabidopsis thaliana* plants (Mouse-ear Cress) were exposed for three days to different U concentrations at pH 7.5. In the roots, the increased capacities of ascorbate peroxidase and glutathione reductase indicate an important role for the ascorbate-glutathione cycle during U-induced stress. However, a significant decrease in the ascorbate redox state was observed after exposure to 75 and 100 µM U, indicating that those roots are severely stressed. In accordance with the roots, the ascorbate-glutathione cycle plays an important role in the antioxidative defence systems in *A. thaliana* leaves exposed to U at pH 7.5 as the ascorbate and glutathione biosynthesis were upregulated. In addition, small inductions of enzymes of the antioxidative defence system were observed at lower U concentrations to counteract the U-induced stress. However, at higher U concentrations it seems that the antioxidative defence system of the leaves collapses as reductions in enzyme activities and gene expression levels were observed.

## 1. Introduction

Uranium (U) is a naturally occurring radionuclide and heavy metal, with an average concentration of 3 mg·kg^−1^ in the earth’s crust [1]. However, large areas have been contaminated with U due to activities such as U mining and milling, metal mining and smelting and the phosphate industry [2]. Uranium causes both a radiation dose and chemical toxicity. However, the chemical toxicity is of greater concern than its radiotoxicity due to the long physical half-life of 4.47 × 10^9^ years, giving U-238 a low specific activity of 1.25 × 10^4^ Bq·g^−1^·U [3].

The mobility and bioavailability of U is dependent on the physicochemical form of U, which is described by the speciation. Important factors controlling the speciation are, for example, pH value, ionic strength, and availability of inorganic and organic ligands [4]. The speciation of U in the Hoagland solution (nutrient solution for *Arabidopsis thaliana* plants) at different pH levels was reported by Saenen, *et al.* [5]. At pH 4.5, UO_2_^2+^ was mainly present, while at pH 7.5, (UO_2_)_2_CO_3_(OH)_3_^−^ was the dominant species. The difference in speciation resulted in remarkable differences in U uptake and translocation in the plant, with high uptake and low translocation at pH 4.5 and lower uptake but higher translocation at pH 7.5. Uranium accumulation and distribution in different plant species has been reported by several authors [6,7,8,9]. However, little information is available on the cellular toxicity of U under contrasted chemical speciation conditions. Toxicity of U would be predominantly caused by UO_2_^2+^ [10,11,12,13]. Trenfield *et al.* [14] investigated the influence of dissolved organic carbon (DOC) on three different Australian tropical freshwater species. By geochemical speciation modeling, they confirmed that the decreased U toxicity that was observed after DOC addition was primarily due to a decrease in UO_2_^2+^ through complexation with DOC. In addition, Markich *et al.* [13] provided evidence that UO_2_^2+^ and UO_2_OH^+^ are the uranium species most responsible for eliciting adverse biological responses to freshwater biota where UO_2_^2+^ has twice the toxic effect of UO_2_OH^+^.

It has been demonstrated that exposing plants to heavy metals can lead to the induction of oxidative stress related responses [5,15,16,17,18]. During oxidative stress, an imbalance between the rate of reactive oxygen species (ROS) production and their degradation will occur [19]. There are different sources of ROS, some of which are involved in normal cell metabolism, such as photosynthesis and respiration [20]. Under stress conditions, ROS production may be enhanced, possibly leading to an abnormal metabolism, loss of physiological functions and death [19]. However, the ROS produced can also act as signals for the activation of stress responses and defence pathways [20]. Important sources of ROS under stress are nicotinamide adenine dinucleotide phosphate (NADPH) oxidases (respiratory burst oxidase homologs, RBOH), and lipoxygenases (LOX) [20,21,22]. To regulate the intracellular concentration of ROS, plant cells evolved an antioxidative defence system consisting of enzymes such as superoxide dismutase (SOD), peroxidases (Px) and catalase (CAT), and antioxidants such as ascorbate (AsA) and glutathione (GSH) [23]. SOD removes superoxide (O_2_^•−^) by catalysing its dismutation to hydrogen peroxide (H_2_O_2_) and O_2_. The intracellular level of H_2_O_2_ is regulated by CAT and Px [24]. Furthermore, the AsA-GSH pathway plays an important role in the antioxidative mechanism in which metabolites and enzymes act together to detoxify H_2_O_2_ [25,26].

Since it is suggested that different U species present at different pH levels can influence U toxicity and the related stress responses, it is important to investigate U effects at different environmentally relevant pH levels. Saenen *et al.* [5] have investigated U uptake, U translocation and some oxidative stress responses after U exposure over a broad pH range. As they only used one U concentration (25 µM), further investigations are needed. While the U-induced effects at pH 4.5 have been investigated before [27,28], the present study focuses on the dose-dependent effects observed after U exposure at high pH. For this purpose, 18-day-old *Arabidopsis thaliana* plants were exposed for three days to different U concentrations ranging from 0 to 100 µM U at pH 7.5.

## 2. Results

### 2.1. Uranium Uptake

**Figure 1:**
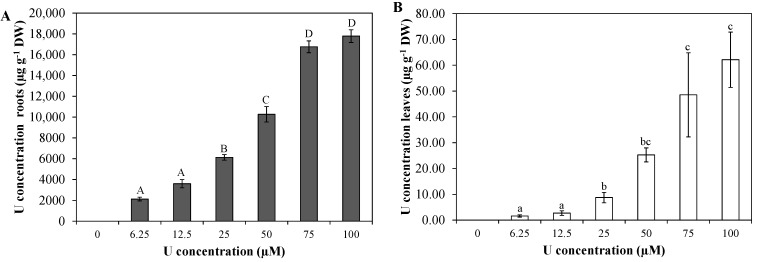
Uranium concentration (µg·g^−1^ DW) in *Arabidopsis thaliana* roots (**A**) and leaves (**B**) exposed to different U concentrations for three days at pH 7.5. Statistical analyses were done separately for leaves and roots. Each point represents the mean ± standard error (S.E.) of at least four biological replicates. Data points with different letters are significantly different (*p <* 0.05).

Exposure of *Arabidopsis thaliana* plants to U for three days resulted in a concentration-dependent increase of the U concentration in the roots and leaves (Figure 1A,B). However, the U concentration in the roots is about 300 times higher than in the leaves, indicating a small root-to-shoot translocation of U.

### 2.2. Growth Responses

**Figure 2:**
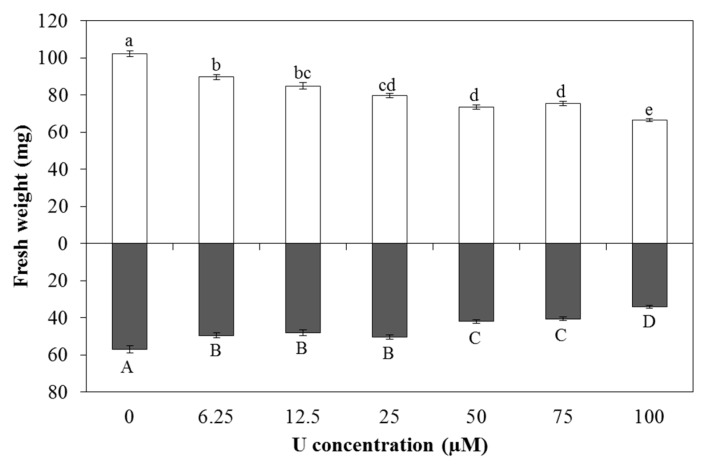
Fresh weight (mg per plant) of roots (grey bars) and leaves (white bars) of *Arabidopsis thaliana* plants exposed to different U concentrations for three days at pH 7.5. Statistical analyses were done separately for leaves and roots. Values represent the mean ± S.E. of at least 100 biological replicates. Data points with different letters are significantly different (*p <* 0.05).

After U exposure, a significant decrease in root and leaf fresh weight was observed (Figure 2). This decrease was already significant after exposure to the lowest U concentration applied (6.25 µM). For the growth reduction in roots and leaves (growth of controls = 100%), a dose response curve was modelled using the four-parameter Weibull function [29,30] in the statistical software package R (version 2.15.0) (R Foundation for Statistical Computing, Vienna, Austria) since this model is the best-fit model for our data. The curve fitting enabled to calculate effect concentrations (ECs) together with a corresponding standard error. The ECx is the concentration that causes × per cent effect compared to a control. For roots exposed for 3 days to U, the EC10, EC30 and EC50 values for root growth reduction were 2.84 ± 1.80, 22.67 ± 6.43 and 70.24 ± 10.48 µM U, respectively. For leaf growth reduction 1.08 ± 0.30, 13.55 ± 1.43 and 53.74 ± 3.51 µM U were calculated to be the EC10, EC30 and EC50, respectively.

### 2.3. Lipid Peroxidation

**Figure 3:**
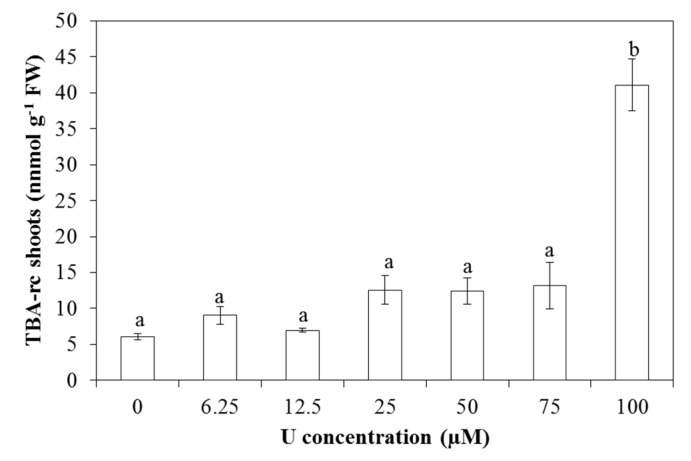
Level of lipid peroxidation, based on the amount of thiobarbituric acid reactive compounds (TBA-rc) in *Arabidopsis thaliana* leaves exposed to different U concentrations for three days at pH 7.5. Values represent the mean ± S.E. of at least four biological replicates. Data points with different letters are significantly different (*p <* 0.05).

The amount of thiobarbituric acid reactive compounds (TBA-rc) was determined as a measure of lipid peroxidation in leaves of U-exposed *Arabidopsis thaliana* plants. A significant increase in lipid peroxidation was observed after exposure to 100 µM U as compared to the control leaves (Figure 3).

### 2.4. Antioxidative Metabolites

To analyse the importance of the AsA-GSH cycle under U stress, the concentrations of both metabolites were determined spectrophotometrically. In the roots, U exposure did not affect total AsA concentrations (Table 1). However, a significant decrease in reduced AsA was observed after exposure to 75 and 100 µM U as compared to the control roots, which was accompanied by a significant increase in dehydroascorbic acid (DHA). Correspondingly, the percentage-reduced AsA decreased with a significantly decrease after exposure to 75 and 100 µM U. For GSH, no significant differences were observed in total and reduced GSH concentrations as compared to the control roots. However, a significant increase in oxidized glutathione (GSSG) was observed after exposure to 50 and 100 µM U, with a corresponding significant decrease in percentage-reduced GSH at 100 µM U.

In contrast to the roots, a significant increase in the total AsA concentration was observed in the leaves after exposure to 12.5 µM U or higher (Table 1). This increase corresponds to a significant increase in reduced AsA with no significant changes in DHA. This led to an increasing trend in percentage-reduced AsA after U exposure. For GSH, an increasing trend in total and reduced GSH was observed, with a significant increase after 12.5, 25 or 50 µM U. However, the total and reduced GSH concentrations after exposure to 75 and 100 µM U were not significantly different as compared to the control. No significant differences were observed in the GSSG concentration or in the percentage reduced GSH (Table 1).

**Table 1:** Ascorbate and glutathione concentrations (nmol·g^−1^ FW) in roots and leaves of *Arabidopsis thaliana* plants exposed to different U concentrations at pH 7.5 for three days. Each point represents the mean of at least four biological replicates ± S.E. Statistical analysis were done separately for leaves and roots. Different capital letters indicate significant differences in the roots (*p <* 0.05). Different small letters indicate significant differences in the leaves (*p <* 0.05). AsA = Reduced ascorbate, DHA = Dehydroascorbic acid, Total AsA = AsA + DHA, % red AsA = Reduced AsA/total AsA, GSH = Reduced glutathione, GSSG = Oxidized glutathione, Total GSH = GSH + GSSG, % red GSH = Reduced GSH/total GSH.

Plant Organ	Metabolite	0 µM U	6.25 µM U	12.5 µM U	25 µM U	50 µM U	75 µM U	100 µM U
ROOTS	Total AsA	587 ± 67 ^A^	657 ± 87 ^A^	803 ± 51 ^A^	603 ± 25 ^A^	782 ± 33 ^A^	688 ± 23 ^A^	680 ± 78 ^A^
	AsA	512 ± 48 ^A,B^	575 ± 92 ^A^	678 ± 55 ^A^	477 ± 48 ^A,B^	530 ± 45 ^A^	230 ± 31 ^B,C^	242 ± 38 ^C^
	DHA	75 ± 26 ^A^	82 ± 20 ^A^	126 ± 17 ^A^	125 ± 43 ^A^	252 ± 44 ^A,B^	368 ± 17 ^B^	439 ± 85 ^B^
	% red AsA	97 ± 6 ^A^	99 ± 7 ^A^	84 ± 2 ^A^	86 ± 9 ^A^	68 ± 5 ^A,B^	47 ± 3 ^B,C^	38 ± 8 ^C^
	Total GSH	120 ± 8 ^A,B^	115 ± 8 ^A,B^	137 ± 6 ^A^	110 ± 3 ^B^	139 ± 5 ^A^	120 ± 4 ^A,B^	121 ± 4 ^A,B^
	GSH	117 ± 7 ^A,B^	113 ± 8 ^A,B^	133 ± 6 ^A^	107 ± 3 ^B^	133 ± 5 ^A^	115 ± 4 ^A,B^	115 ± 4 ^A,B^
	GSSG	1.3 ± 0.4 ^A^	1.3 ± 0.4 ^A^	2.1 ± 0.2 ^A,B^	1.3 ± 0.4 ^A^	2.9 ± 0.3 ^B^	2.4 ± 0.2 ^A,B^	3.0 ± 0.3 ^B^
	% red GSH	98 ± 1 ^A^	98 ± 1 ^A^	97 ± 0.7 ^A,B^	98 ± 0.7 ^A^	96 ± 0.3 ^A,B^	96 ± 0.4 ^A,B^	95 ± 0.4 ^B^
LEAVES	Total AsA	2917 ± 271 ^a^	3752 ± 272 ^a,b^	5041 ± 311 ^b^	4246 ± 414 ^a,b^	5029 ± 265 ^b^	4714 ± 180 ^b^	4940 ± 363 ^b^
	AsA	2676 ± 277 ^a^	3476 ± 261 ^a,b^	4498 ± 286 ^b^	4008 ± 461 ^a,b^	4858 ± 236 ^b^	4570 ± 189 ^b^	4962 ± 346 ^b^
	DHA	241 ± 35 ^a^	276 ± 55 ^a^	543 ± 153 ^a^	238 ± 93 ^a^	170 ± 31 ^a^	145 ± 16 ^a^	248 ± 34 ^a^
	% red AsA	92 ± 1 ^a^	93 ± 1 ^a,b^	89 ± 3 ^a^	94 ± 3 ^a^	97 ± 1 ^b,c^	97 ± 1 ^c^	95 ± 1 ^a,c^
	Total GSH	235 ± 13 ^a^	279 ± 18 ^a,c^	324 ± 41 ^b,c^	322 ± 29 ^b,c^	333 ± 20 ^c^	256 ± 15 ^a,b^	329 ± 60 ^a,c^
	GSH	219 ± 14 ^a^	257 ± 17 ^a,c^	304 ± 38 ^c^	301 ± 28 ^b,c^	305 ± 22 ^c^	234 ± 12 ^a,b^	297 ± 56 ^a,c^
	GSSG	8.0 ± 1.4 ^a^	11 ± 2.9 ^a^	9.8 ± 1.9 ^a^	11 ± 0.9 ^a^	14 ± 1.5 ^a^	11 ± 2.1 ^a^	16 ± 2.1 ^a^
	% red GSH	93 ± 1 ^a^	92 ± 2 ^a^	94 ± 1 ^a^	93 ± 1 ^a^	91 ± 1 ^a^	92 ± 1 ^a^	90 ± 1 ^a^

### 2.5. Enzyme Capacities

**Figure 4:**
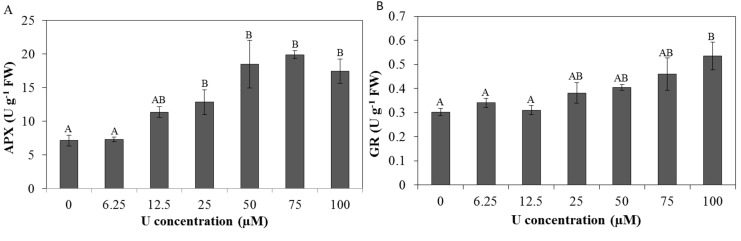
Enzyme capacities (units (U)·g^−1^ FW) of ascorbate peroxidase (**A**, APX) and glutathione reductase (**B**, GR) in *Arabidopsis thaliana* roots exposed to different U concentrations for three days at pH 7.5. Values represent the mean ± S.E. of at least four biological replicates. Data points with different letters are significantly different (*p <* 0.05).

**Figure 5:**
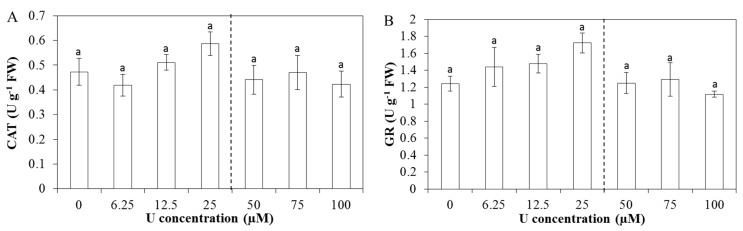

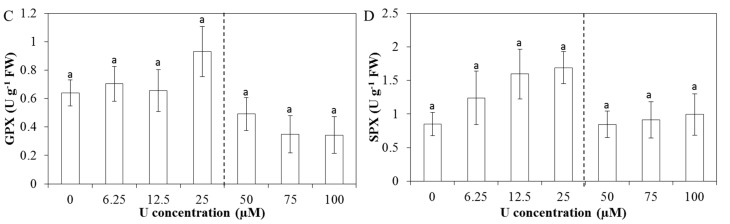
Enzyme capacities (units (U) g^−1^ FW) of catalase (**A**, CAT); glutathione reductase (**B**, GR); guaiacol peroxidase (**C**, GPX) and syringaldazine peroxidase (**D**, SPX) in *Arabidopsis thaliana* leaves exposed to different U concentrations for three days at pH 7.5. Values represent the mean ± S.E. of at least four biological replicates. The vertical line indicates the transition from the increasing trend in enzyme capacities to the decreased capacity. Data points with different letters are significantly different (*p <* 0.05).

Capacities of several enzymes related to the antioxidative defence system were analysed at protein level to investigate their importance under U stress (Figure 4 and Figure 5). In the roots, a significant increase in ascorbate peroxidase (APX) capacity was observed after exposure to 25 µM U or higher as compared to the control plants (Figure 4A). An increasing trend in the capacity of glutathione reductase (GR) was also observed after U exposure, with a significant increase after exposure to 100 µM U (Figure 4B). No significant differences were observed in the capacities of CAT, guaiacol peroxidase (GPX), syringaldazine peroxidase (SPX) and SOD (Table S1).

In the leaves, no significant differences were observed in enzyme capacities after U exposure. However, for CAT, GR, GPX and SPX, an increasing trend in the enzyme capacities was observed up to 25 µM U, after which the activities declined again (Figure 5). This trend was not observed in the SOD and APX capacities (Table S1).

### 2.6. Gene Expression Analysis

The involvement of some ROS-producing and -scavenging enzymes in the oxidative stress responses in *Arabidopsis thaliana* plants exposed to U was evaluated at transcriptional level using quantitative real-time PCR.

First, gene expression of ROS-producing enzymes was analysed in the roots. The plasma membrane bound NADPH oxidases (*RBOHC*/*D*/*F)* are induced in different biotic and abiotic stress conditions as an important source of O_2_^•−^ production [25]. However, in the roots of our plants, a significant decrease in the expression levels of *RBOHC* was observed after exposure to 100 µM U as compared to the control roots (Table 2). Expression levels of *RBOHD*/F remained stable for all treatments. Another source of ROS production are LOX. They catalyse the dioxygenation of polyunsaturated fatty acids, producing hydroperoxy fatty acids [31]. A significant increase in *LOX1* expression was observed in the roots after exposure to 75 µM U.

Concerning gene expression levels of different isoforms of SOD in the roots, *CSD2* (plastidic copper (Cu)/zinc (Zn) SOD) transcript levels decreased significantly as compared to the control roots after exposure to 75 and 100 µM U (Table 2), while a decreasing trend for *CSD1* (cytoplasmatic Cu/Zn SOD) was observed. In contrast, the *FSD1* (plastidic iron (Fe) SOD) expression increased significantly after exposure to 25 µM U or higher. Since it is known that the CSD transcript levels are post-transcriptionally regulated by miRNA398b/c, their expression was measured [32]. A significant increase in the transcript levels of *MIR398b*/*c* was observed after exposure to 100 µM U.

Furthermore, gene expression of enzymes important in H_2_O_2_ scavenging and enzymes related to the AsA-GSH cycle were analysed. In the roots, an increased *CAT1* (peroxisomal CAT) expression was observed after exposure to 100 µM U (Table 2). *GR1* (cytoplasmatic GR) and *GR2* (plastidic GR) expression decreased significantly after exposure to all U concentrations (*GR1*) and 100 µM U (*GR2*). Finally, enzymes involved in GSH and phytochelatin production were analysed at transcriptional level. However, no clear pattern was observed for the *GSH1*, *GSH2* and *PCS1* expression (Table 2).

In the leaves, no significant differences were observed in the ROS-producing enzymes (*RBOHC*/*D*/*F* and *LOX1*/*2*) as compared to the control (Table 3). Concerning the ROS-scavenging mechanisms, a significant decrease in *CSD1*/2 transcript levels was observed after exposure to 25 µM U or higher. As mentioned before, the transcript levels of CSDs are downregulated by microRNA398b/c. As such, a significant increase in *MIR398b*/c expression was observed after exposure to 75 and 100 µM U. In addition, a transient increase in *FSD1* expression was observed up to 25 µM U, after which the expression decreased again, with a significant decrease after exposure to 100 µM U. A significant decrease was also observed in *FSD2* expression after exposure to 100 µM U.

**Table 2:** Relative gene expression levels in *Arabidopsis thaliana* roots of the genes involved in reactive oxygen species (ROS) production and scavenging after exposure to different U concentrations at pH 7.5. Gene expression is expressed relative to the control, which was set to 1. Values represent the mean ± S.E. of at least 3 biological replicates. Significant differences compared to the control plants are indicated with p < 0.01 p < 0.05 for down-regulated and p < 0.01 p < 0.05 for up- regulated genes.

Functional Class	Gene	Roots					
		6.25 µM U	12.5 µM U	25 µM U	50 µM U	75 µM U	100 µM U
Pro-oxidative marker genes	*RBOHC*	0.87 ± 0.14	0.85 ± 0.14	1.08 ± 0.13	0.81 ± 0.05	0.80 ± 0.18	0.23 ± 0.03
	*RBOHD*	1.62 ± 0.30	1.13 ± 0.34	1.24 ± 0.13	1.20 ± 0.13	1.39 ± 0.14	1.07 ± 0.14
	*RBOHF*	1.05 ± 0.08	1.24 ± 0.30	1.12 ± 0.17	1.07 ± 0.23	1.48 ± 0.22	0.98 ± 0.14
	*LOX1*	0.64 ± 0.20	0.62 ± 0.07	0.72 ± 0.07	0.90 ± 0.09	1.92 ± 0.17	1.53 ± 0.22
Anti-oxidative defence marker genes	*CSD1*	1.22 ± 0.11	1.22 ± 0.26	0.91 ± 0.10	0.99 ± 0.08	0.91 ± 0.19	0.61 ± 0.07
	*CSD2*	0.99 ± 0.14	0.85 ± 0.15	0.64 ± 0.05	0.64 ± 0.04	0.49 ± 0.07	0.36 ± 0.03
	*CSD3*	0.43 ± 0.06	0.62 ± 0.03	0.60 ± 0.05	0.49 ± 0.03	0.85 ± 0.14	0.49 ± 0.09
	*FSD1*	1.93 ± 0.02	1.57 ± 0.11	5.21 ± 1.71	5.10 ± 0.23	6.51 ± 13.03	13.03 ± 0.97
	*FSD2*	0.49 ± 0.09	0.56 ± 0.07	0.62 ± 0.06	0.67 ± 0.03	0.78 ± 0.11	0.61 ± 0.10
	*FSD3*	1.23 ± 0.12	0.95 ± 0.16	1.13 ± 0.09	1.38 ± 0.36	1.58 ± 0.37	1.62 ± 0.21
	*MSD1*	2.11 ± 0.14	2.07 ± 0.43	1.71 ± 0.33	1.78 ± 0.23	2.23 ± 0.50	2.40 ± 0.18
Gene expression regulating genes	*miRNA398b*	1.74 ± 0.17	1.00 ± 0.29	1.74 ± 0.25	1.59 ± 0.12	2.59 ± 0.52	3.65 ± 1.04
	*miRNA398c*	1.27 ± 0.22	1.10 ± 0.37	1.89 ± 0.22	1.66 ± 0.16	2.72 ± 0.55	5.29 ± 1.50
Anti-oxidative defence marker genes	*CAT1*	0.90 ± 0.12	1.02 ± 0.18	1.07 ± 0.17	1.06 ± 0.14	0.79 ± 0.24	1.90 ± 0.19
	*CAT2*	1.52 ± 0.18	1.01 ± 0.16	0.95 ± 0.16	1.12 ± 0.14	0.50 ± 0.10	0.62 ± 0.05
	*CAT3*	0.84 ± 0.19	0.73 ± 0.19	1.27 ± 0.38	0.69 ± 0.12	0.77 ± 0.09	0.91 ± 0.25
Genes involved in AsA-GSH cycle	*APX1*	0.93 ± 0.08	1.18 ± 0.18	1.20 ± 0.15	1.23 ± 0.17	1.28 ± 0.13	0.98 ± 0.08
	*GR1*	0.53 ± 0.06	0.42 ± 0.03	0.37 ± 0.06	0.36 ± 0.05	0.48 ± 0.07	0.30 ± 0.01
	*GR2*	0.76 ± 0.10	0.79 ± 0.10	0.99 ± 0.16	0.76 ± 0.13	0.78 ± 0.11	0.52 ± 0.06
Genes involved in GSH and PCs biosynthesis	*GSH1*	0.73 ± 0.05	0.55 ± 0.04	0.67 ± 0.15	0.59 ± 0.09	0.65 ± 0.08	0.52 ± 0.09
	*GSH2*	0.94 ± 0.05	0.73 ± 0.10	0.95 ± 0.16	0.88 ± 0.12	1.24 ± 0.04	0.88 ± 0.15
	*PCS1*	1.04 ± 0.18	0.83 ± 0.06	0.84 ± 0.08	1.24 ± 0.15	1.20 ± 0.08	1.23 ± 0.21

In the expression of H_2_O_2_ scavenging enzymes in the leaves, a significant decrease in the transcript levels of *CAT2* and *APX1* was observed after exposure to 100 µM U (Table 3). The expression of *CAT3* showed a transient increase up to 50 µM U after which the expression declined again. The *GR1* and *GR2* expression in the leaves were significantly decreased after exposure to 12.5 µM U or higher. Finally, a significant decrease in the expression of *GSH1* was observed after exposure to 75 and 100 µM U, while an increasing trend for the *GSH2* expression was observed, with a significant increase after exposure to 100 µM U. No differences were observed in *PCS1* expression.

**Table 3:** Relative gene expression levels in *Arabidopsis thaliana* leaves of the genes involved in ROS production and scavenging after exposure to different U concentrations at pH 7.5. Gene expression is expressed relative to the control, which was set to 1. Values represent the mean ± S.E. of at least 3 biological replicates. Significant differences compared to the control plants are indicated with p < 0.01 p < 0.05 for down-regulated and p < 0.01 p < 0.05 for upregulated genes.

Functional Class	Gene	Leaves					
		6.25 µM U	12.5 µM U	25 µM U	50 µM U	75 µM U	100 µM U
Pro-oxidative marker genes	*RBOHC*	2.44 ± 1.41	2.16 ± 0.57	1.04 ± 0.18	1.11 ± 0.46	0.23 ± 0.05	0.54 ± 0.07
	*RBOHD*	0.79 ± 0.03	1.65 ± 0.34	0.98 ± 0.23	0.91 ± 0.34	0.92 ± 0.20	1.01 ± 0.12
	*RBOHF*	1.34 ± 0.17	1.32 ± 0.19	0.73 ± 0.20	0.56 ± 0.08	0.51 ± 0.13	0.41 ± 0.05
	*LOX1*	0.81 ± 0.16	0.92 ± 0.23	0.44 ± 0.14	0.64 ± 0.15	0.63 ± 0.10	0.55 ± 0.09
	*LOX2*	0.73 ± 0.15	0.90 ± 0.19	0.66 ± 0.22	1.37 ± 0.30	1.88 ± 0.35	1.82 ± 0.10
Anti-oxidative defence marker genes	*CSD1*	0.48 ± 0.12	0.64 ± 0.09	0.36 ± 0.08	0.23 ± 0.05	0.11 ± 0.01	0.05 ± 0.01
	*CSD2*	0.38 ± 0.10	0.43 ± 0.011	0.12 ± 0.02	0.09 ± 0.01	0.04 ± 0.00	0.01 ± 0.00
	*CSD3*	0.52 ± 0.07	0.70 ± 0.14	1.07 ± 0.27	0.86 ± 0.20	0.81 ± 0.11	0.34 ± 0.05
	*FSD1*	1.03 ± 0.20	1.43 ± 0.23	2.59 ± 0.73	1.46 ± 0.12	0.45 ± 0.08	0.28 ± 0.09
	*FSD2*	0.68 ± 0.12	0.83 ± 0.26	0.77 ± 0.27	0.59 ± 0.10	0.42 ± 0.04	0.18 ± 0.04
	*FSD3*	0.79 ± 0.04	1.05 ± 0.26	0.47 ± 0.11	0.72 ± 0.15	0.47 ± 0.05	0.37 ± 0.08
	*MSD1*	0.78 ± 0.08	0.91 ± 0.13	0.75 ± 0.19	0.70 ± 0.15	0.73 ± 0.18	0.46 ± 0.09
Gene expression regulating genes	*miRNA398b*	1.04 ± 0.19	1.49 ± 0.22	0.92 ± 0.05	1.65 ± 0.39	2.38 ± 0.40	2.72 ± 0.47
	*miRNA398c*	1.04 ± 0.18	1.46 ± 0.21	0.74 ± 0.09	1.80 ± 0.36	2.84 ± 0.44	2.79 ± 0.53
Anti-oxidative defence marker genes	*CAT1*	0.85 ± 0.13	0.84 ± 0.26	1.33 ± 0.51	0.65 ± 0.47	0.68 ± 0.24	0.50 ± 0.16
	*CAT2*	0.91 ± 0.18	0.42 ± 0.14	1.03 ± 0.38	0.58 ± 0.08	0.34 ± 0.08	0.16 ± 0.05
	*CAT3*	0.67 ± 0.10	1.25 ± 0.24	2.17 ± 0.50	1.98 ± 0.53	1.07 ± 0.08	0.78 ± 0.27
Genes involved in AsA-GSH cycle	*APX1*	0.98 ± 0.04	1.06 ± 0.08	0.77 ± 0.21	0.59 ± 0.13	0.61 ± 0.12	0.41 ± 0.02
	*GR1*	0.92 ± 0.05	0.67 ± 0.03	0.54 ± 0.09	0.48 ± 0.07	0.49 ± 0.02	0.46 ± 0.03
	*GR2*	0.75 ± 0.09	0.76 ± 0.08	0.58 ± 0.08	0.53 ± 0.11	0.43 ± 0.08	0.29 ± 0.03
Genes involved in GSH and PCs biosynthesis	*GSH1*	0.89 ± 0.06	1.04 ± 0.09	1.17 ± 0.19	0.88 ± 0.12	0.57 ± 0.07	0.46 ± 0.03
	*GSH2*	1.23 ± 0.09	1.33 ± 0.12	1.29 ± 0.13	1.53 ± 0.17	1.65 ± 0.24	1.80 ± 0.25
	*PCS1*	1.52 ± 0.26	1.73 ± 0.30	1.28 ± 0.29	0.89 ± 0.19	1.17 ± 0.24	1.08 ± 0.10

## 3. Discussion

The present study aims to investigate the dose-dependent effects of U exposure at pH 7.5 in order to elucidate the mechanisms that are involved after U exposure at high pH. Therefore, 18-day-old *Arabidopsis thaliana* plants were exposed to different U concentrations ranging from 0 to 100 µM U for three days at pH 7.5.

Roots are in direct contact with the nutrient solution, which resulted in a dose-dependent increase in the U concentration of the roots. This increase was accompanied by a significant reduction in root fresh weight, which was already observed after exposure to the lowest U concentration applied (6.25 µM) (Figure 2). For root growth reduction, the EC10, EC30 and EC50 were calculated using the four-parameter Weibull function [29,30]. The observed EC50 (70.24 ± 10.48 µM U) was remarkably higher than the value observed for root growth reduction at pH 4.5 (28.14 ± 1.59 µM U) [28]. This can possibly be related to the fact that at acidic pH, U is more easily taken up by the roots which will lead to a higher U content in the roots and as such to a faster decrease in root growth. In addition, also the differences in U speciation can contribute to the discrepancy in EC50 values. At pH 4.5, there was mainly the presence of UO_2_^2+^, while at pH 7.5 the carbonate species were mainly present. Since UO_2_^2+^ is suggested to be the most toxic U species, this can possibly contribute to the fact that more toxic effects were observed at low pH.

In the leaves, a dose-dependent increase of U was also observed. However, the U concentration in the leaves was about 300 times lower than in the roots, indicating a low root-to-shoot translocation factor ranging between 3.5 × 10^−3^ and 7.7 × 10^−4^, depending on the U concentrations added. A small root-to-shoot translocation of U has been reported before [5,11,33,34]. Despite the low U concentrations in the leaves, a significant decrease in leaf fresh weight was observed (Figure 2). The EC50 (53.74 ± 3.51 µM U) is comparable to the EC50 observed by Horemans *et al.* [35] for leaf growth reduction after U exposure at pH 5.5. They observed an EC50 value of 66 µM U. However, the EC50 for reduction in leaf growth observed after U exposure at pH 4.5 was remarkably lower (27.13 ± 5.20 µM U) [5]. Since the U translocation at pH 7.5 is higher than at pH 4.5, resulting in a higher leaf U content at pH 7.5 following exposure to the same nominal U concentration, this indicates that U is more toxic in the leaves after exposure to U at acidic pH. The large differences in U uptake by the roots, with high uptake at pH 4.5 and lower uptake at pH 7.5, can possibly contribute to the decreased growth in the leaves at low pH due to root-to-shoot signalling. An important role for root-to-shoot signalling under U stress was suggested previously by Vanhoudt *et al.* [15].

Generally, under stress conditions, the generation of toxic ROS species is increased [36]. Since U is a redox-active metal, it can directly induce ROS formation non-enzymatically through Fenton and Haber-Weiss reactions. Another important source of ROS are the membrane bound NADPH oxidases as they catalyse the formation of O_2_^•−^ [25]. Gene expression of some important NADPH oxidases was analysed. However, no induction of *RBOHC/D/F* was observed in the roots (Table 2). This indicates that the NADPH-mediated oxidative burst is probably not involved in the ROS production in the roots after U exposure at pH 7.5. These results are partially in agreement with the results of Vanhoudt *et al.* [37]. They observed a significant induction of the *RBOHD* expression after exposure to 100 µM U at pH 5.5, but no increased expression of *RBOHC* and *RBOHF*. In addition, the NADPH-mediated oxidative burst was also not involved in the roots of *Arabidopsis thaliana* plants exposed to U at pH 4.5 [28]. LOX can also lead to the production of ROS such as singlet oxygen and O_2_^•−^. A transient dose-dependent induction of *LOX1* was observed in roots after U exposure, with a significant induction after exposure to 75 µM U (Table 2), which is in agreement with the results of Vanhoudt *et al.* [15], who reported an increased *LOX1* expression in *Arabidopsis thaliana* roots exposed to 100 µM U at pH 5.5. In the leaves of *Arabidopsis thaliana* plants exposed to U at pH 7.5, it seems that the NADPH oxidases and LOX are not involved in the U-induced stress responses since no significant differences were observed in their gene expression levels (Table 3.

To counteract the toxicity of the U-induced oxidative stress, plants possess an antioxidative defence system composed of ROS-scavenging enzymes (e.g., SOD, CAT, APX) and antioxidative metabolites (AsA and GSH) [38]. Concerning ROS-scavenging enzymes, SOD constitutes the first line of defence by dismutating O_2_^•−^ to H_2_O_2_. SODs are present at different subcellular locations. Depending on the metal co-factor that is used, different isoforms of SOD can be distinguished [39]. At enzymatic level, no induction of the SOD capacity in the roots was observed. However, at transcriptional level, a shift in the expression of the different SOD isoforms was noticed. As such, a significant reduction in *CSD2* expression was observed after exposure to 75 and 100 µM U. Since the miRNA398b/c targets *CSD1* and *CSD*2 [40], the decreased expression of *CSD2* can be related to the induction of *MIR398b*/c after exposure to 75 and 100 µM U, as was shown before being under U stress at low pH [28]. As the CSDs are dispensable, the loss of *CSD2* can be compensated by an increased *FSD1* expression, which is observed after exposure to 25 µM U or higher. As such, the scavenging of O_2_^•−^ can be maintained in the plastids. Yamasaki *et al.* [41] proposed that miRNA398 is involved in the regulation of Cu homeostasis since *MIR398b/c* contains Cu-sensitive GTAC motifs in its promoter region. Under conditions where Cu is limited, the expression of miRNA398b/c will be upregulated, which will lead to a downregulation of the Cu-requiring CSDs. Although the Cu content in the roots of our plants was not decreased (Table S2), a decreasing trend in the Cu content in the leaves was observed at the higher U concentrations (Table S2), as was observed before by Vanhoudt *et al.* [33]. By decreasing the expression of the CSDs in the roots, more free Cu will be available in the roots which can be transported to the plastocyanins, where it is an essential element in photosynthetic electron transport in higher plants. Therefore, Cu will probably be saved for the most essential functions during limited Cu availability [42].

In the roots, the AsA-GSH cycle probably plays an important role in the scavenging of H_2_O_2_ under U stress as indicated by an increased capacity of both APX and GR (Figure 4). APX has a high affinity for H_2_O_2_ and is able to tightly control the H_2_O_2_ concentrations, rendering it the ideal candidate to control the H_2_O_2_ levels for signalling [16]. For the scavenging of H_2_O_2_, APX needs AsA as a reductant. During the reaction, AsA will be oxidized to DHA. GSH is used as reducing substrate to reduce DHA back to AsA by DHA reductase. During this reaction, GSH will be oxidized to GSSG, which in turn will be re-reduced to GSH. This reaction is catalysed by GR [43]. The increased GR capacity observed after U exposure indicates that the roots try to keep GSH in its reduced state to ensure DHA reduction to AsA. However, the increased GR activity is not sufficient to maintain AsA in its reduced state, since only 38% reduced AsA was present after exposure to 100 µM U (Table 1). Since DHA accumulation is considered as a negative event for cell metabolism [44], this indicates that the roots exposed to 50 µM U or higher at pH 7.5 are highly stressed, which was also indicated by the decreased fresh weight.

Like in the roots, the AsA-GSH pathway also plays an important role in the antioxidative defence mechanisms in the leaves. In contrast to the roots, there was no induction of APX and GR capacity but a significant increase in the total AsA and GSH concentration after U exposure (Table 1). This indicates an enhanced capacity to detoxify H_2_O_2_. An increase in the antioxidative metabolites after heavy metal exposure has been observed before by Vanhoudt *et al.* [15]. In addition, exposing a cell culture of *Brassica napus* to 50 µM U resulted in a significant increase in GSH concentration [45]. Finally, Aranjuelo *et al.* [46] observed in glutathione deficient *Arabidopsis mutants* (*cad2.1*) the highest MDA and least reduced ascorbate levels, which highlights the importance of GSH under U stress. However, in the present study, the increase in total GSH was only transient, with a significant increase after exposure to 12.5–50 µM U but no significant differences after exposure to 75 and 100 µM U. This can be related to the decreased GSH biosynthesis as suggested by a decreased expression of *GSH1* that was observed at the two highest U concentrations (Table 3). GSH1 codes for γ-glutamylcysteine synthetase. As this enzyme is responsible for the rate-limiting step in GSH production [47], a decreased expression can lead to a decreased synthesis of GSH. Those results are in agreement with Saenen *et al.* [27]. They observed a decrease in the transcript levels of *GSH1* and *GSH2* in leaves of *Arabidopsis thaliana* plants exposed to U at pH 4.5, leading to a decrease in the total GSH concentration. In addition to the transient increase in the GSH concentration in the present study, transient increases in several enzyme capacities and gene expression levels were observed. As such, a small (non-significant) induction was observed in the CAT, GR, GPX and SPX capacity up to 25 µM U (Figure 5). At higher U concentrations, the enzyme activities declined again. At the gene expression level, the *CAT3* and *FSD1* expressions are also slightly upregulated, up to 50 µM U after which their expression declined (Table 3). This biphasic response can possibly indicate that at lower U concentrations, leaves are able to defend themselves against U-induced oxidative stress by upregulating the antioxidative defence mechanisms (*i.e.*, increasing trend in enzyme activities and increased biosynthesis of AsA and GSH), while at higher U concentrations the leaves can no longer cope with the U-induced stress and the defence mechanisms collapse.

Similar to the roots, miRNA398b/c is involved in the regulation of the *CSD1*/2 expression in the leaves. A significant increase in *MIR398b/c* expression was observed after exposure to 75 and 100 µM U, accompanied by a decreased *CSD1*/*2* expression in leaves of plants exposed to 50 µM U or higher. As such, the miRNA398b/c response seems to be a general U response, since similar results were observed in the leaves of plants exposed to U at pH 4.5 [27]. In contrast to the roots, this decrease in *CSD1*/2 was not compensated by an increased *FSD1* expression at higher U concentrations (Table 3), as was also observed in the leaves exposed to U at pH 4.5 [27]. Vanhoudt *et al.* [33] reported before that U exposure at pH 5.5 disturbed the nutrient uptake and distribution of several nutrients in *Arabidopsis* seedlings. They observed a significant decrease in leaf Fe content after exposure to 100 µM U for 3 days, which is in accordance with the results of the present study (Table S2). The decreased Fe content can explain the lack of compensation by *FSD1* at higher U concentrations, since under Fe limiting conditions the *FSD* expression can decline [48]. This will result in decreased synthesis and subsequent sequestration of Fe into these proteins. The decreased expression of both *CSD1/2* and *FSD1/2* can lead to a decreased capacity to scavenge O_2_^•−^ in the plastids, which in turn can lead to an enhanced stress level.

## 4. Experimental Section

### 4.1. Plant Culture and Treatment

*Arabidopsis thaliana* seeds (Columbia ecotype) were surface sterilized and incubated in the dark for three days at 4 °C on moist filter paper to synchronize germination. Seeds were sown on plugs from 1.5 mL polyethylene centrifuge tubes filled with 0.6% agar in Hoagland solution with low phosphate content [18]. The plugs were positioned in a PVC cover capable of holding 36 plugs. Next, the cover was placed on a container filled with 1.35 L of a modified Hoagland solution with a pH of 5.5 (1 mM KNO_3,_ 0.3 mM Ca(NO_3_)_2_, 0.2 mM MgSO_4_, 0.1 mM NH_4_H_2_PO_4_, 1.62 µM FeSO_4_, 0.78 µM EDTA, 4.6 µM H_3_BO_3_, 0.9 µM MnCl_2_, 32 nM CuSO_4_, 55.6 nM H_2_MoO_4_, 76.5 nM ZnSO_4_). Plants were grown in a growth chamber (Microclima 1000E, Snijders Scientific B.V., Tilburg, The Netherlands) under a 14 h photoperiod (photosynthetic photon flux density of 150 µmol·m^−2^·s^−1^ at the leaf level, supplied by Sylvania BriteGro F36WT8/2084 and F36WT8/2023), with day/night temperatures of 22 °C/18 °C and 65% relative humidity. After 18 days preculture, the pH of the nutrient solution was adjusted to pH 7.5 with NaOH and HCl. To retain the pH at a constant level, 500 µM MES (2-(*N*-morpholino)ethanesulfonic acid) and 500 µM TRIS (tris(hydroxymethyl)-aminomethane) were added. Plants were exposed to 0, 6.25, 12.5, 25, 50, 75 or 100 µM U. Uranium was added as UO_2_(NO_3_)_2_.6H_2_O (SPI chemicals, West Chester, PA, USA) from a 100 mM stock solution to the Hoagland nutrient solution. Since roots can exudate organic acids or anions [49], the pH of the nutrient solution was adjusted twice a day. During the exposure time, a modified Hoagland solution was used with 0.025 mM NH_4_H_2_PO_4_ [18]. After three days of exposure, plants were harvested. Leaf and root fresh weight was determined and samples were snap frozen in liquid nitrogen and stored at −80 °C. Leaf and root growth was determined as (fresh weight_day 21_ − fresh weight_day 18_)/(fresh weight_control day 21_ − fresh weight_control day 18_)] × 100.

### 4.2. Uranium and Nutrient Analysis

Root and leaf samples were taken for the determination of U, Fe and Cu. Roots were washed twice for 10 min in 1 mM Pb(NO_3_)_2_ and once for 10 min with distilled water to exchange surface-bound U, Fe or Cu. Afterwards, root and leaf samples were dried for at least one week at 70 °C. The oven-dried samples were calcinated in a muffle furnace at 550 °C. After cooling down to room temperature, the plant material was digested into 1 M HCl. The U-238 concentration in these samples was determined by using a quadrupole inductively coupled plasma-mass spectrometer (ICP-MS) (XSeries II, Thermo Scientific, Bremen, Germany) equipped with a PFA-ST Nebulizer (Elemental Scientific, Omaha, NE, USA) and a peltier cooled (2 °C) cyclonic quartz spray chamber for sample introduction. Calibration curves were established using U standard solutions (0 to 10 μg·L^−1^) prepared from a single element stock solution (SPEX Industries Inc., Edison, NJ, USA). The instrumental detection limit for U was 2 ng·L^−1^. Typical precision for samples with U concentrations well above the limit of detection was below 5% (relative standard deviation, 10 replicates). The concentration of Cu and Fe were determined by a ICP-optical emission spectrometry (ICP-OES, 710 Series, Agilent Technologies, Dieghem, Belgium).

### 4.3. Determination of Lipid Peroxidation

The thiobarbituric acid reactive compounds (TBA-rc) were used as a measure for membrane damage. Approximately 120 mg of leaves were homogenized in 1 mL 0.1% trichloroacetic acid (TCA, Sigma-Aldrich, Saint Louis, MO, USA) using an ice-cold mortar and pestle. After centrifugation at 20,000× *g* for 10 min, 250 µL supernatant was diluted with 1 mL TBA/TCA solution (0.5% TBA (Sigma-Aldrich, Saint Louis, MO, USA) in 20% TCA). The mixture was 30 min incubated at 95 °C and quickly cooled down in an ice bath. After another centrifugation step of 10 min at 20,000× *g*, the absorbance of the supernatant was determined spectrophotometrically at 532 nm and corrected for the non-specific absorbance at 600 nm [50]. The content of TBA-rc was calculated according to the law of Lambert-Beer (ε = 155 mM^−1^·cm^−1^) taking into account the fresh weight and the dilutions made.

### 4.4. Metabolite Measurements

Oxidized and reduced forms of ascorbate and glutathione were measured spectrophotometrically using a plate-reader assay. Approximately 80 mg of root samples or 120 mg of leaf samples were ground in liquid nitrogen and then extracted in 600 µL (roots) or 800 µL (leaves) HCl. Measurements were done as described by Queval and Noctor [51] with following modifications. For the measurement of total ascorbate levels (reduced ascorbate (AsA) + dehydroascorbic acid (DHA)), 100 µL supernatant was incubated with 25 mM DTT in a 120 mM NaH_2_PO_4_ (pH 7.5) buffer for 15 min at 20 °C to convert DHA to AsA. Hereafter, the pH of the incubated supernatant was adjusted to pH 5.5, the optimal pH for ascorbate oxidase (AO). Other measurements were performed as described by Queval and Noctor [51].

### 4.5. Analysis of Enzyme Capacities

Approximately 100 mg frozen leaf or root tissue was homogenized in 0.1 M Tris-HCl buffer (pH 7.8) containing 1 mM EDTA, 1 mM DTT and 4% insoluble polyvinylpyrrolidone (PVP) using a mortar and pestle. The homogenate was squeezed through a nylon mesh and centrifuged at 20,000× *g* and 4 °C for 10 min. The enzyme capacities were determined spectrophotometrically in the supernatant at 25 °C as described before by Vanhoudt *et al.* [18]. For the determination of the APX capacity, an extraction buffer was used which contained 10 mM sodium-AsA since APX loses stability in the absence of AsA, which will lead to a declined activity of the enzyme [52].

### 4.6. Gene Expression

Frozen leaf or root tissue (maximum 100 mg) was disrupted in 2 mL microcentrifuge tubes under frozen conditions using steel beads (diameter 3 mm) and the Retsch Mixer Mill MM400. RNA was extracted using the RNeasy Plant Mini kit (Qiagen, Venlo, The Netherlands) according to the manufacturer’s instructions. The RNA quantity and integrity were determined spectrophotometrically at 230, 260 and 280 nm (Nanodrop, Isogen Life Science, De Meern, The Netherlands) and via gel electrophoresis (Bioanalyzer, Agilent Technologies, Santa Clara, CA, USA), respectively. Before cDNA synthesis, RNA samples were incubated in gDNA wipeout buffer at 42 °C for 2 min to remove contaminating genomic DNA. First strand cDNA synthesis was primed with a combination of oligo(dT)-primers and random hexamers according to the manufacturer’s instructions using QuantiTect Reverse Transcription Kit (Qiagen, Venlo, The Netherlands). Equal amounts of starting material were used (1 µg). Quantitative PCR was performed with the ABI Prism 7500 (Applied Biosystems, Foster City, CA, USA), using SYBR Green chemistry. PCR amplifications were performed at universal cycling conditions (10 min 95 °C, 40 cycles of 15 s at 95 °C and 60 s at 60 °C) in a total volume of 10 μL, containing 2.5 μL cDNA sample, 5 μL Fast SYBR Green Master Mix (Applied Biosystems, Foster City, CA, USA), 0.3 μM forward primer, 0.3 µM reverse primer and 1.9 μL RNase-free H_2_O. Primers used for gene expression analyses are given in Table S3. The amplification efficiencies of the primers were calculated according to Wong and Medrano [53] by making a 4-fold serial dilution over 4 dilution points of a mixed sample and were accepted when they were greater than 80%. Multiple reference genes were used for leaf (*At4g26410*, *At5g25760*, *At5g55840*, *At4g34270*) and root normalization (*At2g28390*, *At5g08290*, *At5g15710*, *At4g05320*, *At4g26410*, *At5g25760*, *At5g55840*, *At4g34270*). The expression stability of the reference genes was evaluated by geNorm (version 3.5) [54]. Gene expression data were calculated relative to the control treatment following the 2^−ΔΔ*C*t^ method [55], normalized to a normalization factor based on the expression level of multiple reference genes.

### 4.7. Statistical Analysis

Statistical analyses were done separately for leaves and roots. Uranium effects were determined by one-way ANOVA using the free software package GNU R (version 2.15.0) (R foundation for Statistical Computing, Vienna, Austria). Statistical differences for in-group means were determined after Tukey adjustment for multiple comparisons. Normal distribution of the data was tested using Shapiro-Wilk test. Logarithmic or square root transformations were applied where necessary to obtain normal distribution of the data. If the assumption of normality was not fulfilled, a non-parametric Wilcoxon rank sum test was carried out. Homoscedasticity was evaluated by the Bartlett’s test.

## 5. Conclusions

U exposure at high pH resulted in a significant reduction in leaf and root fresh weight, which already was observed after exposure to the lowest U concentration applied (6.25 µM). However, EC50 values for root and leaf growth reduction were higher at pH 7.5 as compared to pH 4.5, possibly indicating that U causes more toxic effects at low pH. Although the increased APX activity indicates an enhanced detoxification of H_2_O_2_, the increased GR capacity was not sufficient to keep AsA in its reduced state. The concomitant reduction in the AsA redox state indicates that the roots are seriously stressed after exposure to 75 and 100 µM U. However, the increased APX and GR activity point towards an important role for the AsA-GSH cycle in the root defence mechanisms after U exposure. In the leaves, the AsA-GSH cycle also plays an important role, as indicated by an increased biosynthesis of both AsA and GSH. While at low U concentrations the leaves seemed capable to defend themselves against oxidative stress, the antioxidative defence system collapsed at higher U concentrations with a reduction in enzyme capacities and a decreased gene expression of several enzymes. Finally, the decreased expression of *CSD1/2* in the leaves was not compensated by an increased *FSD1/2* expression at higher U concentrations. This can indicate a decreased capacity to scavenge O_2_^·−^ in the plastids, which, in turn, can lead to an oxidative challenge.

## Supplementary Materials

Supplementary materials can be found at http://www.mdpi.com/1422-0067/16/06/12405/s1.
