# Supplementary material for: Induction of Oxidative Stress and Antioxidative Mechanisms in Arabidopsis thaliana after Uranium Exposure at pH 7.5
Source: Int J Mol Sci. 2015 Jun 2;16(6):12405–23. doi: 10.3390/ijms160612405 (PMC4490451; doi:10.3390/ijms160612405)
Supplement: Supplementary file 1 [file ijms-16-12405-s001.pdf]

Supplementary Information

Table S1. Enzyme capacities (units g^{-1} FW) of superoxide dismutase (SOD), catalase (CAT), guaiacol peroxidase (GPX) and syringaldazine peroxidase (SPX) of *Arabidopsis thaliana* roots and SOD and ascorbate peroxidase (APX) capacities of *Arabidopsis thaliana* leaves exposed to different U concentrations for 3 days at pH 7.5. Values represent the mean ± S.E. of at least 4 biological replicates. Data points with different letters are significantly different (p < 0.05).

Organ	Enzyme	0 μM U	6.25 μM U	12.5 μM U	25 μM U	50 μM U	75 μM U	100 μM U
ROOTS	SOD	124 ± 14 ^{A}	130 ± 19 ^{A}	135 ± 25 ^{A}	123 ± 23 ^{A}	99 ± 12 ^{A}	154 ± 26 ^{A}	128 ± 21 ^{A}
	CAT ($\times 10^{-3}$)	45 ± 8 ^{A}	66 ± 10 ^{A}	57 ± 11 ^{A}	81 ± 9 ^{A}	54 ± 8 ^{A}	77 ± 12 ^{A}	60 ± 4 ^{A}
	GPX	8.9 ± 0.9 ^{A}	12.58 ± 1.3 ^{A}	12.7 ± 1.6 ^{A}	14.7 ±1.2 ^{A}	13.1 ± 1.5 ^{A}	15.9 ± 1.7 ^{A}	17.0 ± 3.2 ^{A}
	SPX	0.57 ± 0.21 ^{A}	1.33 ± 0.38 ^{A}	0.78 ± 0.19 ^{A}	1.81 ± 0.36 ^{A}	1.07 ± 0.15 ^{A}	1.24 ± 0.44 ^{A}	1.12 ± 0.47 ^{A}
LEAVES	SOD	134 ± 7 ^{a}	159 ± 12 ^{a}	154 ± 25 ^{a}	159 ± 12 ^{a}	153 ± 17 ^{a}	171 ± 23 ^{a}	169 ± 12 ^{a}
	APX	11.4 ± 1.6 ^{a}	15.3 ± 2.8 ^{a}	16.7 ±3.1 ^{a}	13.8 ± 2.3 ^{a}	10.5 ± 1.4 ^{a}	16.8 ± 2.1 ^{a}	14.2 ± 3.2 ^{a}

Table S2. Element concentrations (mg g^{-1} DW) in roots and leaves of Arabidopsis thaliana seedlings after exposure to different uranium concentrations for 3 days. Results represent mean ± S.E. of at least 3 biological replicates. Data points with different letters are significantly different ($p < 0.05$). N/A: metal content in plants exposed to 0 µM U was not determined. n.d.: not detectable. *: value based on one measurement.

Organ	Element	0 µM U	6.25 µM U	12.5 µM U	25 µM U	50 µM U	75 µM U	100 µM U
ROOTS	Fe	N/A	495.83 ± 41.80 ^{AB}	441.59 ± 74.31 ^{A}	516.85 ± 48.47 ^{AB}	685.25 ± 43.26 ^{B}	418.44 ± 14.57 ^{AB}	409.41 ± 20.63 ^{AB}
	Cu	N/A	n.d.	n.d.	19.21 ± 0.82 ^{A}	29.87 ± 1.89 ^{B}	40.47 ± 1.12 ^{C}	41.62 ± 0.74 ^{C}
LEAVES	Fe	N/A	49.58 ± 1.68 ^{a}	48.40 ± 2.39 ^{a}	48.17 ± 1.64 ^{a}	45.60 ± 0.73 ^{ab}	41.32 ± 1.22 ^{b}	41.63 ± 1.48 ^{b}
	Cu	N/A	6.50 ± 0.41 ^{a}	6.95 ± 0.72 ^{a}	6.85 ± 0.18 ^{a}	5.71 ^{a*}	n.d.	n.d.

Table S3. Sequences of forward and reverse primers used in gene expression analysis.

Locus	Gene	Forward Primer	Reverse Primer
**Reference Genes**			
At2g28390	*SAND family protein*	AACTCTATGCAGCATTTGATCCACT	TGATTGCATATCTTTATCGCCATC
At4g26410	*expressed*	GAGCTGAAGTGGCTTCCATGAC	GGTCCGACATACCCATGATCC
At4g34270	*TIP41-like*	GTGAAAACTGTTGGAGAGAAGCAA	TCAACTGGATACCCTTTCGCA
At5g08290	*Mitosis protein YSL8*	TTACTGTTTCGGTTGTTCTCCATTT	CACTGAATCATGTTCGAAGCAAGT
At5g15710	*F-box protein*	TTTCGGCTGAGAGGTTCGAGT	GATTCCAAGACGTAAAGCAGATCAA
At5g25760	*UBC*	CTGCGACTCAGGGAATCTTCTAA	TTGTGCCATTGAATTGAACCC
At5g55840	*PPR gene*	AAGACAGTGAAGGTGCAACCTTACT	AGTTTTTGAGTTGTATTTGTCAGAGAAAG
At4g05320	*UBQ10*	GGCCTTGTATAATCCCTGATGAATAAG	AAAGAGATAACAGGAACGGAAACATAGT
**Other Genes**			
At1g07890	*APX1*	TGCCACAAGGATAGGTCTGG	CCTTCCTTCTCTCCGCTCAA
At1g20630	*CAT1*	AAGTGCTTCATCGGGAAGGA	CTTCAACAAAACGCTTCACGA
At4g35090	*CAT2*	AACTCCTCCATGACCGTTGGA	TCCGTTCCCTGTCGAAATTG
At1g20620	*CAT3*	TCTCCAACAACATCTCTTCCCTCA	GTGAAATTAGCAACCTTCTCGATCA
At1g08830	*CSD1*	TCCATGCAGACCCTGATGAC	CCTGGAGACCAATGATGCC
At2g28190	*CSD2*	GAGCCTTTGTGGTTCACGAG	CACACCACATGCCAATCTCC
At5g18100	*CSD3*	GTTGTTGTGCATGCGGATCC	CACATCCAACTCTCGAGCCTG
At4g25100	*FSD1*	CTCCCAATGCTGTGAATCCC	TGGTCTTCGGTTCTGGAAGTC
At5g51100	*FSD2*	TTGGAAAGGTTCAAGTCGGCT	CATTTGCAACGTCAAGTCTATTCG
At5g23310	*FSD3*	AACGGGAATCCTTTACCCGA	TGTCTCCACCACCAGGTTGC
At3g24170	*GR1*	CTCAAGTGTGGAGCAACCAAAG	ATGCGTCTGGTCACACTGC
At3g54660	*GR2*	GCCCAGATGGATGGAACAGAT	TAGGGTTGGAGAATGTTGGCG
At4g23100	*GSH1*	CCCTGGTGAACTGCCTTCA	CATCAGCACCTCTCATCTCCA
At5g2738	*GSH2*	GGACTCGTCGTTGGTGACAA	TCTGGGAATGCAGTTGGTAGC
At1g55020	*LOX1*	TTGGCTAAGGCTTTTGTCGG	GTGGCAATCACAAACGGTTC
At3g10920	*MSD1*	ATGTTTGGGAGCACGCCTAC	AACCTCGCTTGCATATTTCCA
At5g44070	*PCS1*	TGGTGTTGAATGCTCTTTCTATCG	GGTTCGCAGCAATCCAACAT
At5g14545	*pri-miRNA398b*	AGTAATCAACGGCTGTAATGACGCTAC	TGACCTGAGAACACATGAAAACGAGAG
At5g14565	*pri-miRNA398c*	TCGAAACTCAAACTGTAACAGTCC	ATTTGGTAAATGAATAGAAGCCACGGGCCACG
At5g51060	*RBOHC*	TCACCAGAGACTGGCACAATAAA	GATGCTCGACCTGAATGCTC
At5g47910	*RBOHD*	TATGCATCGGAGAGGCTGCT	TAGAGACAACACGTTCCCGGG
At1g64060	*RBOHF*	GGTGTCATGAACGAAGTTGCA	AATGAGAGCAGAACGAGCATCA
